# Iconicity and Sign Lexical Acquisition: A Review

**DOI:** 10.3389/fpsyg.2017.01280

**Published:** 2017-08-02

**Authors:** Gerardo Ortega

**Affiliations:** ^1^Centre for Language Studies, Radboud University Nijmegen, Netherlands; ^2^Max Planck Institute for Psycholinguistics Nijmegen, Netherlands

**Keywords:** iconicity, sign language, L1 acquisition, L2 acquisition, degree of iconicity, form-meaning

## Abstract

The study of iconicity, defined as the direct relationship between a linguistic form and its referent, has gained momentum in recent years across a wide range of disciplines. In the spoken modality, there is abundant evidence showing that iconicity is a key factor that facilitates language acquisition. However, when we look at sign languages, which excel in the prevalence of iconic structures, there is a more mixed picture, with some studies showing a positive effect and others showing a null or negative effect. In an attempt to reconcile the existing evidence the present review presents a critical overview of the literature on the acquisition of a sign language as first (L1) and second (L2) language and points at some factor that may be the source of disagreement. Regarding sign L1 acquisition, the contradicting findings may relate to iconicity being defined in a very broad sense when a more fine-grained operationalisation might reveal an effect in sign learning. Regarding sign L2 acquisition, evidence shows that there is a clear dissociation in the effect of iconicity in that it facilitates conceptual-semantic aspects of sign learning but hinders the acquisition of the exact phonological form of signs. It will be argued that when we consider the gradient nature of iconicity and that signs consist of a phonological form attached to a meaning we can discern how iconicity impacts sign learning in positive and negative ways.

## Introduction

The view that languages consist solely of linguistic labels with arbitrary relations to their referents is no longer held. Iconic words, whose linguistic forms emulate perceptual, sensori-motor characteristics of a referent (Perniss et al., 2010), are highly prevalent in many languages of the world. Research on non-Western languages has convincingly demonstrated that iconicity is not limited to onomatopoeias like *woof-woof, miaow,* or *moooh*, but rather stretches to a large number of linguistic forms such as ideophones, phonaesthemes, and mimetic verbs (Assaneo et al., 2011; Dingemanse, 2012). Further, recognition that human communication is multimodal in nature (i.e., it uses hands, eye-gaze, and other bodily cues) has given further evidence that iconicity is critical during face-to-face interaction (Vigliocco et al., 2014), particularly when we consider the gestures produced by speakers (McNeill, 1992; Kendon, 2004). The relevance of iconicity becomes more palpable when we look at the sign languages used by the deaf communities where a large proportion of their linguistic structures are motivated by the form of their referent (Klima and Bellugi, 1979; Cuxac, 1999a,b; Taub, 2001; Pietrandrea, 2002; Cuxac and Sallandre, 2007; Pizzuto et al., 2007). Overall, the Saussurian view that the relationship between words and the concept they represent is exclusively arbitrary has fallen out of favor and currently it is widely recognized that iconicity is an equally important design feature of language (Perniss et al., 2010; Perniss and Vigliocco, 2014; Dingemanse et al., 2015).

One of the current aims of the multimodal study of language is to explain the impact of iconic structures in language processing and language acquisition. Regarding the latter, it has been argued that iconic forms assists in solving the problem of referentiality because they fit more faithfully to the perceptual features of the referent allowing language learners to isolate a referent from a crowded scene and link it to a linguistic label (Imai and Kita, 2014; Perniss and Vigliocco, 2014). The facilitating effect of iconicity in word learning has been widely demonstrated in many spoken languages with populations of different ages (e.g., Imai et al., 2008; Herold et al., 2011; Kantartzis et al., 2011; Revill et al., 2014; Lockwood et al., 2016). When looking at the gestures that accompany speech, it has been widely documented that iconic manual forms have a positive effect in word learning across a range of age groups (Tellier, 2008; Kelly et al., 2009; Macedonia and von Kriegstein, 2012; de Nooijer et al., 2014; Macedonia and Klimesch, 2014). Together these studies support the view that iconicity – both in speech and gesture – has a positive effect in word learning not only in infants but also in adult learners across different cultural groups.

A more complex picture emerges when we look at sign languages. Traditional accounts suggest that iconicity does not facilitate vocabulary development in deaf children acquiring a sign language from birth because they lack the world knowledge and cognitive maturity to make form-referent associations (Orlansky and Bonvillian, 1984; Newport and Meier, 1985; Meier et al., 2008). These claims received further support from studies showing that it is not until after the age of three that toddlers are capable of making form-meaning associations (Namy, 2008; Tolar et al., 2008). More recently, however, the role of iconicity has been revisited with studies showing that the first signs acquired by deaf children are iconic (Thompson et al., 2013) and that type of iconicity is relevant in sign development (Tolar et al., 2008; Ortega et al., 2014, 2017). Further, some studies investigating the acquisition of a sign language as a second language report a positive effect in hearing non-signers (Lieberth and Gamble, 1991; Campbell et al., 1992; Baus et al., 2012; Morett, 2015). However, there is also contradicting evidence reporting that iconicity may in fact hinder some aspects of sign learning (Ortega and Morgan, 2015a,b). Given the growing interest in iconicity and linguistic development in both modalities of language (oral-aural and manual-visual), it is paramount to assess the opposing findings in the sign literature and establish points of convergence and divergence.

The present review article will focus on two domains of sign learning at the lexical level. First, it will describe empirical studies exploring the role of iconicity in learners of a sign language as first language (L1); i.e., by deaf children learning sign from their parents. It will be explained that the contradictory findings may be attributed to iconicity being operationalised as a blanket term when a more fine-grained definition may show an effect in sign-acquiring children, in particular, if we focus on signs with the most direct mappings (i.e., absolute iconicity, Dingemanse et al., 2015). The second part will focus on hearing adults learning a sign language as a second language (L2). This section will explain that there is a clear dissociation in the effect of iconicity in that it facilitates conceptual-semantic aspects of sign learning but hinders the acquisition of the exact phonological form of signs.

This review is divided as follows. The first section describes how sign languages incorporate iconicity while being constrained by a conventionalised linguistic system. The next section gives a general description of the main components of word learning, and importantly, explains how learners allocate resources to acquire the formal and semantic aspects of a new linguistic label. Crucially, this section will highlight that certain paradigms assess only one aspect of word learning (i.e., form or meaning) and as such caution should be made regarding claims on iconicity and sign learning. The review then moves on to describe empirical studies showing positive and negative effects in sign acquisition in L1 and L2 learners. It will then be suggested that the acquisition of other signed structures (e.g., morphological markers and classifier constructions) may also be susceptible to the effect of iconicity in sign learning. The article concludes by summarizing the literature reviewed and will propose mechanisms explaining the effect of iconicity in sign learning.

Before delving into the studies on iconicity and sign learning, it is important to explain how iconicity is expressed in the manual-visual modality and the linguistic nature of signed systems.

## Sign Iconicity and Linguistic Constraints

**FIGURE 1:**
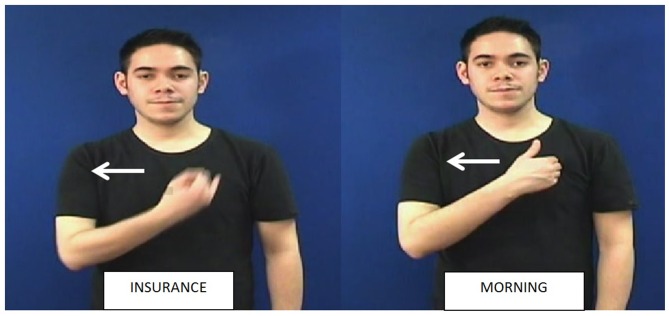
Minimal pairs in British Sign Language (BSL). Both signs share the same location [torso] and movement [left-right] but INSURANCE is articulated with the handshape 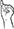 and morning with the handshape 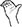.

One of the most important linguistic discoveries of the 20th century is that the manual languages used by the Deaf communities are fully fledged languages with the same expressive power as spoken languages (Stokoe, 1960). Sign languages are not universal and their grammatical structures are independent from the grammars of the surrounding speaking community. For instance, American Sign Language (ASL) and British Sign Language (BSL) are unrelated sign languages unintelligible between them and follow a different grammar from spoken English. Importantly, sign languages have been found to have the same organizational principles as spoken languages; i.e., phonology, morphology, lexicon, semantics (Sandler and Lillo-Martin, 2006). Sign languages have the property of phonology, that is, sub-lexical units combine to create a meaningful sign. The minimal features that constitute a sign are the configuration of the hand (handshape), its coordinates with respect to a plane (orientation), its position in signing space (location), and the trajectory or internal hand transitions occurring during the execution of a sign (movement) (Stokoe, 1960; Brentari, 1999; van der Kooij, 2002). These sub-lexical units are critical to define a sign and modification of any of its components results in a different sign. In the same way that replacing a phoneme of a word can change its meaning (e.g., /**m**at/ vs. /**b**at/), modification of one of the sign components results in a different sign (see **Figure 1** for an example of a minimal pair in British Sign Language). The phonological repertoire of each sign language has a finite number of elements and they may vary significantly cross-linguistically. Acquisition of these sub-lexical units is paramount because they play a key role during lexical access and sign processing (Conlin et al., 2000; Dye and Shih, 2006; Baus et al., 2008; Carreiras et al., 2008; Gutiérrez et al., 2012).

The first scholars investigating sign languages set out to describe their structural organization and used spoken languages as template of the structures that *should* be present in signs. In an effort to convince the world that sign languages were real conventionalised systems, linguists ascribed to the categories and analyses used in spoken languages and downplayed in different degrees those features that did not fit into any linguistic category of speech. Influenced by the Saussurian view that arbitrariness is the holy grail of real languages (de Saussure, 1916), a large number of scholars neglected the relevance of iconicity as a prominent characteristic of sign languages. Despite this dominant view, another wave of academics highlighted the importance of iconicity (Cuxac, 1999a,b; Cuxac and Sallandre, 2007; Pizzuto et al., 2007). Cuxac (1999b), one of the most prominent advocates of iconicity in sign languages, proposes that it shapes sign language discourse at every level and as a result signs can represent physical aspects of a referent, its spatial location on a three-dimensional space, motion patterns, and temporal reference (similar claims have been made by others, e.g., Mandel, 1977; Klima and Bellugi, 1979; Taub, 2001; Pietrandrea, 2002; Cuxac and Sallandre, 2007). Iconic and arbitrary signs are observable in the vocabularies of all sign languages, but it is undeniable that signs that are iconically motivated are ubiquitous in the vocabularies of all signed lexicons. It has been argued that at least a third of all lexical signs are iconic (Boyes-Braem, 1986) and that between 50 and 60% of signs’ structure can be directly linked to the physical features of their referents (Pietrandrea, 2002).

**FIGURE 2:**
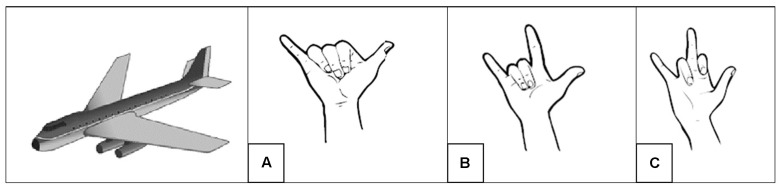
Lexical signs PLANE in BSL **(A)**, American Sign Language **(B)**, and Korean Sign Language **(C)**. The three languages represent iconically the fuselage of a plane but they use distinct phonological handshapes.

Sign iconicity has two important characteristics. First, despite being visually motivated by the visual-spatial characteristics of a referent, signed structures are constrained by phonotactic, language-specific principles. For instance, the BSL sign PLANE consists of the handshape 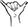 moving across signing space, and represents the fuselage of an airplane. The sign PLANE in ASL and Korean Sign Language (KSL) also represent the fuselage of a plane but differ in the handshape to represent it (**Figure 2**). This goes to show that even when sign languages resort to similar strategies to represent a referent iconically, they have linguistic conventions not necessarily shared across languages.

**FIGURE 3:**
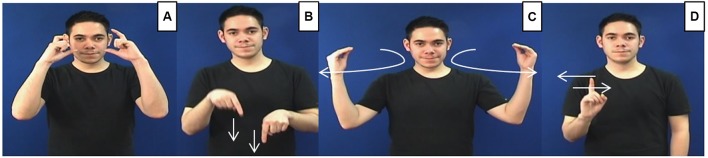
British Sign Language (BSL) signs showing different degrees of meaning transparency. Transparent signs are the easiest to relate to their referent (e.g., **A**: CAMERA), followed by translucent (e.g., **B**: TO-LIMP), then obscure signs (e.g., **C**: HOLLAND), and the most difficult to associate with its concept are opaque signs (e.g., **D**: WHAT).

The second important characteristic is that iconicity is not a categorical property of signs but rather lies within a continuum with some signs being easier to link with their referent than others. Klima and Bellugi (1979) proposed four levels of sign iconicity, each representing different degrees of access to their meaning to non-signers. *Transparent* signs are the most evident and easy to link to a referent even when presented in isolation (e.g., the sign CAMERA). *Translucent* signs are those whose meaning might not be immediately clear but people may still be able to pick some of the aspects represented by the sign (e.g., TO-LIMP). *Obscure signs* also have a link with their referent but the ordinary observer may be able to understand the connection only after the connection is explained (e.g., HOLLAND represents the traditional Dutch bonnet). Finally, *opaque signs* are those without an evident connection with their referent (e.g., WHAT) (**Figure 3**). More recently, Emmorey (2014) has put forward the notion of Iconicity as Structure mapping which suggests that a sign (i.e., phonological form) may overlap in varying degrees with a conceptual representation, and that the effect of iconicity will be observed only in the most iconic forms (e.g., signs representing handling depictions).

The graded nature of iconicity has important implications for sign acquisition in that the form-meaning link of some signs may be evident to most individuals but some others are more difficult to access. Additionally, the capacity to access the iconic properties of signs depends not only on their intrinsic properties but also on the cultural background and age group of the perceiver. When asked to guess the meaning of iconic signs in Italian Sign Language (LIS) hearing non-signers belonging to the same culture (i.e., Italian non-signers) perform better than hearing individuals from a different culture because some iconic signs refer to aspects that can only be understood by people sharing the same background (Grosso, 1993; Pizzuto and Volterra, 2000). Similarly, hearing non-signing children (6 years of age) produce different iconicity ratings for some iconic signs than deaf signers and hearing non-signers because they lack the conceptual knowledge depicted in a sign (e.g., the ASL sign DOCTOR recreates the action of checking the pulse on the wrist, a practice possibly unfamiliar to many children) (Griffith et al., 1981). Therefore, iconicity should not be considered a blanket term that applies equally to all signs. Comprehension of iconicity goes beyond resemblance between a linguistic form and a referent because it is heavily reliant on a number of factors grounded in human experience (e.g., age, linguistic experience, cultural background). The notion of degree of iconicity and how it may influence acquisition echoes research in spoken languages showing that some iconic words are more strongly related to their referent than others and as a result they are learnable with different ease (Dingemanse et al., 2016).

After having explained how sign structures map in different degrees to their referent while being constrained by linguistic conventions, the following sections gives a brief description of the components involved in word (and sign) learning so as to get a good understanding of how iconicity may influence sign acquisition in L1 and L2 learners.

## What’S in a Word?

A significant amount of psycholinguistic research has been devoted to the understanding of language production, and more precisely, what are the constituents of a word. This work has led to several proposals aiming to describe the lexical components involved from planning to execution of a linguistic utterance, the information each one encodes, and the number of stages engaged in the process. Despite some differences, the most influential models suggest that lexical access recruits sequentially two components. One level encodes semantic information about a concept whereas the other encodes its phonological/orthographic form (Dell and O’Seaghdha, 1992; Caramazza, 1997; Levelt et al., 1999)^1^. That is, lexical items consist of a semantic representation and this is linked to its phonological representation. Research has shown that these two levels are not exclusive to speech because separate semantic and phonological representations have also been attested for sign languages (Baus et al., 2008; Baus and Costa, 2015). Despite being expressed through different channels (aural-oral vs. manual-visual), speech and sign consist of two separate but interdependent levels (semantics and phonology) which constitute our knowledge of a word.

During lexical learning, these two components do not come together as a package but rather are the result of a process which requires developing phonological and semantic representations (Nation, 2001). The cognitive resources to attend to the semantics of a new lexical item compete with resources to attend its phonological form (and vice versa), and one frequently overrides the other (VanPatten, 1990, 1996; VanPatten and Cadierno, 1993; Barcroft, 2004, 2015). In order to evaluate the factors involved in word (and sign) learning, it is critical to understand that semantic-phonological representations are the result of a two-stage process that is often dissociated (Barcroft, 2004, 2015). Many studies use the blanket term ‘word learning’ without explicitly stating that their manipulations measure just one aspect of lexical development. For instance, studies using phoneme monitoring show that newly learnt non-words become fully integrated in the mental lexicon and compete for lexical selection with pre-existing words (e.g., Tamminen and Gaskell, 2008; Dumay and Gaskell, 2015) but actually, these words are just novel phonological structures deprived of meaning. Similarly, forced-choice paradigms require participants to choose from two options the translation equivalent of newly learnt words in an L2 (e.g., Nygaard et al., 2009). However, this task is informative about the semantic aspect of word learning but does not provide any evidence of how the phonological form of the word is acquired.

The distinction between evaluating the form or the meaning of a novel word has important implications in sign acquisition research, especially, to understand whether the observed effects of iconicity relate to the phonological or semantic aspects of sign learning. Signs consist of a (manual) phonological structure linked to semantic representations so it is possible to assume that sign learners are also likely to channel their attentional resources to one of these constituents, in a similar way as has been shown in spoken words (VanPatten, 1990, 1996; VanPatten and Cadierno, 1993; Barcroft, 2004, 2015). As will be argued in the following sections, iconicity seems to facilitate the semantic aspects of sign learning, and not their phonological structure.

It has been explained thus far, first, that iconic structures in sign languages are built upon conventionalised linguistic principles; and second, that sign/word learning consists of the acquisition of a linguistic form attached to a meaning. With this literature as foreground, this review turns to the empirical evidence and assess whether or how iconicity influences lexical development. Regarding sign L1 acquisition, it will be explained that the opposing evidence may be due to the fact that studies frequently interpret iconicity as a categorical property and not as a graded feature that can be accessed in different degrees by different populations. Regarding sign L2 learners, it will be explained that certain experimental paradigms evaluate the phonological *or* semantic aspects of sign learning. By teasing these two components apart it will be possible to get a better understanding of the positive or negative effect of iconicity in sign learning by hearing adults.

## Iconicity and Sign Learning

### Sign L1 Learning

Historical views on symbolic development argued that the acquisition of arbitrary labels stems from children’s capacity to master iconic symbols first (Piaget, 1962). Under this account, children develop the ability to make non-iconic association between a symbol and its referent because they use iconic mappings as initial stepping stone. This position fell out of favor after a large body of evidence showed that it is around their third birthday that children show evidence of understanding direct symbol-referent mappings (Namy, 2004, 2008; Tolar et al., 2008; Suanda et al., 2013). At 3 years of age, children already master a large vocabulary so this led to the conclusion that iconicity is not instrumental for lexical development. Similar claims were made in the sign language literature: most investigations exploring sign lexical development before the age of three show that iconicity does not assist sign acquisition (however, see Thompson et al., 2013).

Within this age window, Orlansky and Bonvillian (1984) examined longitudinal sign production of 13 children of deaf parents during the initial stages of their linguistic development (0;10-0;18 months of age). Caregivers kept a diary in which they included every new sign attempted by their child, the age when it was produced, and the accuracy of articulation. All signs were then classified into iconic (they clearly resembled its referent), metonymic (the sign represents a minor feature of the referent), and arbitrary (there was no similarity between sign and referent). After comparing the proportion of signs produced across participants it was found that there was an equal proportion of signs in the three categories implying that all types of signs are learnt at the same rate. Newport and Meier (1985) proposed that children exposed to a sign language from birth lack the world knowledge that could help them interpret the connection between sign and referent (e.g., the sign MILK in ASL refers to the action of milking a cow which is knowledge that an infant has not yet acquired).

Also looking at lexical development by deaf toddlers (0;08-12;05), Meier et al. (2008) analyzed the signs produced by four deaf children acquiring ASL from birth and investigated whether they produced signs in citation form or whether they exaggerated signs’ iconic properties. The authors argued that if deaf children understand the iconicity of a sign they would exaggerate its features during naturalistic interactions. For example, the iconic sign ICE-CREAM could have an adult-like form (i.e., a closed fist moving toward the mouth with circular movements) or it could introduce a stronger iconic element (e.g., sticking the tongue out to lick one’s hand). The signs produced by children were rated by the researchers to determine whether iconicity was enhanced, reduced, or it remained neutral. The study reports that signs were predominantly produced neutrally with very weak hints of having an exaggerated gestural element suggesting that iconicity is inaccessible to children and thus cannot aid acquisition. Two questions that spring to mind, however, are whether exaggeration of signs’ iconic features could be used as proxy for children’s access to iconicity; and whether production (form) without comprehension (meaning) reveals the influence of iconicity in sign learning.

Some have argued that the striking parallels in lexical development between deaf and hearing children also speaks against the role of iconicity in sign language development. Anderson and Reilly (2002) created the first MacArthur Bates Communicative Development Inventory (CDI) for ASL. The CDI is a checklist of words adapted to many spoken and signed languages in which parents document the productive and receptive skills of their children (i.e., whether they can produce and/or understand certain words/signs). The authors collected longitudinal data from 34 deaf children (0;10-0;36) and compared patterns with that of hearing children learning English. They found that the sequence and developmental trajectories of most linguistic structures in ASL go hand in hand with English. The CDI has also been adapted to BSL (Woolfe et al., 2010), and even though this study does not make a direct comparison between the lexical development of deaf and hearing children, it shows the typical learning trajectory of the CDI in which receptive skills precede productive ability. A different study measuring directly sign acquisition gives further evidence that deaf children follow the same developmental path as hearing children. It has been shown that deaf children acquiring Italian Sign Language understand and produce the same sign equivalents of the words produced by age-matched hearing children (Rinaldi et al., 2014). Both groups of children share the same environment, they talk about the same objects and actions with their caregivers, and thus children learn words and signs based on their everyday occurrence and not because iconicity boosts acquisition. Together, these studies argue that if deaf and hearing children follow almost overlapping developmental trajectories, the same underlying cognitive mechanism is responsible for lexical acquisition – regardless of linguistic modality – and iconicity is not a key player.

Criticisms of these studies are twofold: First, they do not take into consideration parental input or signs’ degree of iconicity. These two points are critical given recent evidence showing that they may play key roles in sign language learning. Regarding caregivers’ input, Perniss et al. (2017) found that when deaf adults interact with an (imaginary) child addressee they tend to modify iconic signs (enlargement, lengthening and repetition) more often than arbitrary signs, in particular with absent referents (with referent present, pointing dominates parental communicative strategies). Regarding signs’ degree of iconicity, recent studies show that iconicity has a positive correlation between age of acquisition and degree of iconicity, even at an age when iconicity is not readily available to deaf children (younger than 3 years). Thompson et al. (2013) used parental reports of the MacArthur Bates BSL CDI to investigate the type of signs acquired by deaf children at the earliest stages of development. The authors collected reports from 31 deaf children between the ages of 0;08-2;06 years of age and compared the signs to previously collected iconicity ratings (Vinson et al., 2008). It was found that for younger and older infants, the first signs produced and comprehended were iconic, even when phonological complexity was factored out. In a more recent study on ASL, Caselli and Pyers (2017) analyzed the ASL-CDI data reported earlier (Anderson and Reilly, 2002) and correlated a set of signs with iconicity ratings, neighborhood density, and lexical frequency. They replicated the findings in BSL in that iconicity facilitates sign language acquisition, but they also report that the other two variables also contribute to lexical development. They conclude that iconicity is a factor that facilitates sign acquisition but in addition, deaf children monitor signs’ phonological properties and frequency of occurrence and leverage them to acquire signs. These recent studies challenge established views on iconicity and posit that while factors like phonological complexity, familiarity, frequency, and neighborhood density also play a role (Thompson et al., 2013; Caselli and Pyers, 2017), iconicity is a key feature that assists learning in deaf children.

More recently, and looking at children older than 3-years of age, Ortega et al. (2014, 2017) argue that type of iconicity matters in the acquisition of a sign language. They looked at lexical development in Turkish Sign Language (*Turk Isaret Dili,* TİD) in signs that have two possible iconic variants for the same concept: one describing an action associated with the referent (e.g., the action sign BED represents a person lying on a pillow) and the other one depicts its perceptual features (e.g., the perceptual sign BED represents a mattress and headboard). Two groups of children (mean ages: 5;02 and 7;02, respectively), two groups of parents and a separate group of adults took part in a picture description task in which they had to explain the spatial configuration between two objects to an interlocutor (e.g., PILLOW-UNDER-BED). Children and adults described the picture to an adult, and critically, parents described the pictures to their own children. After analyzing the proportion of action and perceptual signs produced in all descriptions, it was found that children produce mostly action variants (80%) while adults interacting with other adults produced mainly perceptual variants (approximately 20% of action variants). Interestingly, parents interacting with their children produced roughly the same proportion of action and perceptual variants, but significantly more action signs than adults interacting with other adults. The authors argue that children favor action signs because they can be easily mapped with their motor schemas; and that parents also use them more because they accommodate to their children’s linguistic output.

To sum up, early research on the acquisition of a sign language as L1 suggested that iconicity does not facilitate lexical development because children lack enough conceptual schemas to make associations between a linguistic form and its referent. However, the null effect of iconicity may relate to critically small samples, inconsistencies in parental reports, and crucially, to the way in which researchers measured access to iconicity by deaf children (e.g., exaggerating signs’ iconic instantiation; Meier et al., 2008). Understanding the iconic motivation of signs varies significantly depending on age, linguistic experience, and cultural background (Klima and Bellugi, 1979; Griffith et al., 1981; Pizzuto and Volterra, 2000) so establishing the degree of iconicity of a sign requires of an objective independent measure such as iconicity ratings by deaf participants (e.g., Thompson et al., 2013) or clear operationalisations of sub-types of iconicity. As such, the notion of degree and type of iconicity is critical to determine whether it plays or not a role in sign L1 acquisition. It is possible that weak iconicity does not necessarily play a role in early sign L1 acquisition, but more direct mappings between form and meaning may show an effect (e.g., the sign TO-DRINK may be easier to learn because it has direct correspondences with its referent).

### Sign L2 Learning

Lieberth and Gamble (1991) carried out one of the first empirical attempts to understand whether iconicity influenced sign acquisition by hearing adults. In their experiment, they asked hearing non-signers to see a series of ASL signs along with their spoken and written English equivalents. Signs consisted of a balanced number of iconic and arbitrary signs as determined by the iconicity ratings from a different study (Griffith et al., 1981). Participants were shown the same set of signs 10 min and 2 weeks after the first presentation, and were asked to write down their meaning in English. The English translations of both iconic and arbitrary signs were recalled with the same degree of accuracy after a short delay (10 min). However, after a 1-week delay, iconic signs were recalled significantly better than arbitrary ones. This study is one of the first to recognize that iconicity gives access to a ‘functional, receptive core vocabulary’ to sign naïve hearing adults and thus is an important factor that facilitates L2 vocabulary learning.

Campbell et al. (1992) presented consecutively two lists of BSL signs with varying degrees of iconicity to hearing non-signers and intermediate signers. The task required participants to determine whether the signs in the second list were being shown for the first time or whether they had been presented in the first list. Both groups of participants recalled iconic signs more accurately than arbitrary ones regardless of their prior linguistic experience with BSL. The authors then calculated the number of instances in which participants could and could not name a sign and found that ease of naming strongly correlated with iconicity; i.e., the more iconic the sign, the more likely it was to be named. The study concludes that linguistic experience with a manual language is not a strong predictor for sign recognition but rather degree of iconicity. The authors argue that iconic signs can be dually coded by means of a verbal and a visual representation (Paivio, 1986) and as such they can be more easily recalled and named. They claim that iconic signs have *configural coherence* in that the elements that constitute them have a ‘natural’ structure and their relative organization with one another creates a unified whole.

The benefits of iconicity were further attested in a translation task. Baus et al. (2012) recruited a group of hearing non-signers and taught them a set of iconic and arbitrary signs. After the learning phase, participants took part in a translation recognition task in which they were simultaneously presented with a sign and an English word. They were then required to determine whether sign-word pairs were translations of each other by pressing yes/no keys. Non-signers were faster and more accurate at recognizing iconic than arbitrary signs. A different group of L2 hearing proficient signers carried out the same task (without the training phase) and, surprisingly, they recognized iconic sign pairs more slowly than arbitrary signs. In a follow-up forward translation task, participants were shown the English word on a computer screen and they had to produce the sign equivalent while reaction times were recorded by press release. In a backward translation, participants were shown individual signs and had to produce the English translation while reaction times were recorded from voice onset (i.e., from the moment they produced the first phoneme of their spoken translation). The results show that in both the forward and backward tasks, non-signers were faster at translating iconic than arbitrary pairs. Hearing proficient signers, in contrast, showed no effect of iconicity in the forward translation task, while in the backward translations iconic signs were translated more slowly than arbitrary ones. The authors conclude that iconicity allows non-signers to match a linguistic form with its referent more easily and thus aids memorisation at the early stages of sign language learning. The effect of iconicity is evident in both directions of the translation which further strengthens the claim that iconicity taps in the conceptual system that links an English word and its novel signed equivalent. Iconicity shows a negative effect in hearing proficient signers because iconic signs appear to have more possible meanings, and as such a denser neighborhood leads to higher lexical competition during translation. A negative effect of iconicity due to lexical density has also been reported in a priming study with hearing proficient signers but only for those iconic signs that depict perceptual features of a referent (e.g., signs depicting the outline of a window or the shape of a pair of wings) (Ortega and Morgan, 2015c). While iconicity seems to help hearing non-signers at the earliest stages of sign learning, other linguistic processes such as lexical competition and sign frequency may come into effect in hearing signers with an established manual lexicon.

More recently, and having as foreground theories of embodied cognition (Barsalou, 2008), Morett (2015) investigated the role of iconicity in L2 learning. In a sign learning study, hearing non-signers were taught iconic, arbitrary and metaphoric signs (i.e., those depicting a concrete object but whose meaning relate to an abstraction of the representation, for example, two pointing fingers approaching each other’s tips for the sign GOAL). Signs were presented visually along their English translation in four different conditions. In the enactment condition, participants were required to imitate the sign after presentation of the stimuli. In the visualization condition, they were asked to create a mental image of the referent. In the hand motion condition, they produced a meaningless movement previously shown by the researcher. Finally, in the viewing condition, they saw the sign twice. During the testing phase, which took place 5 min, one and 4 weeks after training, participants were presented with an English word and they had to produce the sign equivalent. Responses were coded as incorrect if participants did not recall the sign or if at least one of signs’ sub-lexical components (i.e., handshape, location, movement and orientation) was ‘notably deviant’ from the target. The results show that signs were recalled more accurately 5 min after presentation than after 1 and 5 weeks. Signs learned in the enactment condition were recalled significantly better than in any other condition. There was no difference in recall between iconic, arbitrary and metaphoric signs. Regarding accuracy in sign production, there was no effect of learning condition but there was an effect of time of testing and type of sign. Arbitrary and metaphoric signs were produced the most accurately after 5 min of training but over time accuracy decreased. In contrast, iconic signs were the most accurately produced at all points in time. The author concludes that embodiment, visual imagery, and iconicity play a positive role in sign L2 learning both in sign recall (i.e., meaning) and production (i.e., form). She claims that visualization aids learning if non-signers’ mental image is compatible with sign iconicity. She also argues that learners devote more attention to the phonology of iconic signs which results in higher articulation accuracy.

The commonality between these studies is that they all find a facilitating effect of iconicity in sign L2 learning (Lieberth and Gamble, 1991; Campbell et al., 1992; Baus et al., 2012; Morett, 2015). It must be noted, however, that with one exception (Morett, 2015) these studies evaluate the effect of iconicity in only one dimension of sign learning; namely the aspect that relates to its meaning. Reaction times by button press, forced-choices, and cued recall in participants’ first language are informative on how conceptual knowledge is more easily retrieved when iconicity is involved, but reveal little information about how accurately participants acquire the phonological structure of signs. More intriguingly are the positive effects in sign production (i.e., form) reported by in Morett’s (2015) study. Potential caveats of its experimental design are, first, that signs were not balanced for phonological complexity across condition; and second, that sign accuracy was measured under a very lax coding scheme (e.g., a sign location would be incorrect if it was articulated at ‘neck level instead of stomach level’ (Morett, 2015; p. 260). One must be cautious about extending the positive effects of iconicity to all aspects of sign learning given that most of the aforementioned studies point that it influences only the conceptual-semantic domain it taps onto. In fact, there is a growing body of evidence suggesting that iconicity hinders the acquisition of formal aspects of signs.

Ortega and Morgan (2015a) investigated the development of a manual phonological system in hearing adults learning BSL as L2. Participants were required to imitate as accurately as possible a set of iconic and arbitrary signs balanced for phonological complexity across conditions. Participants were tested once before they started their BSL course and then after 11 weeks of instruction. The results revealed that iconic signs were articulated significantly less accurately than arbitrary signs. Moreover, when iconic signs consisted of more phonological features (i.e., higher complexity), articulation accuracy decreased accordingly. Learners showed improvement with instruction but the negative effect of iconicity in sign articulation persisted even after 11 weeks of lessons.

**FIGURE 4:**
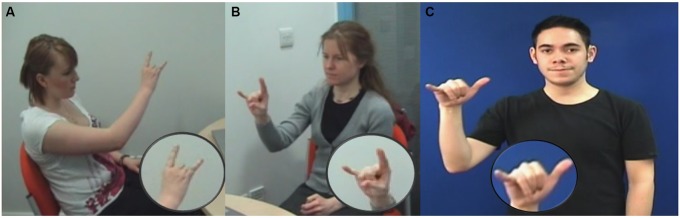
Participants **(A,B)** attempting to produce the BSL sign PLANE **(C)** during a sign repetition task. Participants failed to produce the target handshape yet they executed a sign that retained its iconic motivation.

Ortega and Morgan (2015b) replicated their findings with two different groups of hearing non-signers. In their experiment, the two groups took part in another sign repetition task with the same set of iconic and arbitrary signs described above. One group was presented with BSL signs in isolation while the second group was given the English translation before viewing the sign. The idea was that the English prime could potentially activate conceptual knowledge and somewhat influence sign articulation. Both groups articulated iconic signs less accurately than arbitrary signs regardless of whether they had prior exposure to the English translation of the sign. The results of these two studies were interpreted as evidence that iconicity gives access to the meaning of a sign and thus learners are less attentive to its exact phonological structure. As a consequence, participants reproduced a sign that retained its iconic motivation, but not its exact phonological structure (**Figure 4**). The authors also raise the possibility that learners’ iconic gestures may be interfering in sign articulation because they share similar forms and meaning but no sub-lexical structure (this has also been reported in learners of ASL; Chen Pichler, 2009, 2011). Arbitrary signs, in contrast, could not be tracked to a referent so participants paid more attention to the formal properties of the sign. This interpretation is compatible with research showing that when deaf signers are asked to make phonological judgements about signs (e.g., whether they consist of curved or straight fingers) they are slower at responding to iconic than arbitrary signs (Thompson et al., 2010). Iconic signs give more automatic access to the meaning, so it is cognitively taxing to make form-based judgements.

In sum, evidence thus far shows that iconicity does not aid all aspects of sign L2 learning but rather that there is dissociation between the conceptual-semantic features of a sign and its linguistic structure, with iconicity showing a positive effect on the former, but a negative effect on the latter. On one hand, studies show that iconic signs are recalled and named more accurately than arbitrary signs at the initial stages of sign L2 learning (Lieberth and Gamble, 1991; Campbell et al., 1992; Baus et al., 2012). This positive effect has been interpreted as iconic signs being semantically and imagistically rich sources of information about the referent that strengthen the conceptual link with newly learnt signs. On the other hand, studies directly assessing how iconicity affects the acquisition of the formal aspect of signs (i.e., their phonological structure) clearly show a negative effect in sign production (with the exception of Morett, 2015). Iconicity gives direct access to the meaning of a sign making it less relevant to pay attention to its linguistic conventions. In executing iconic signs, learners at the earliest stages tend to produce signs that encode their iconic instantiation but without their linguistic conventions (Ortega and Morgan, 2015a,b). Taken together these studies suggest that iconicity aids in the acquisition of the semantic-conceptual aspects of sign learning but hinders in learning its specific linguistic conventions.

## Does Iconicity Help?

Linguistic and cognitive sciences continue to amass evidence that iconicity and arbitrariness are fundamental components of language with each of them playing critical yet distinct roles in many language processes (Perniss et al., 2010; Perniss and Vigliocco, 2014; Vigliocco et al., 2014; Dingemanse et al., 2015). It is therefore baffling that sign languages, which excel for their high prevalence of iconic structures, present a complex and sometimes contradicting picture regarding the role of iconicity in L1 and L2 acquisition. The aim of this review article has been to describe the empirical evidence accumulated over the last decades to evaluate how sign language scholars have investigated the role of motivated forms in lexical development (see **Table 1**). The following sections aim to reconcile the contradicting evidence and explain how iconicity operates in L1 and L2 sign lexical acquisition.

**Table 1:** Summary of studies investigating the role of iconicity in sign L1 and L2 acquisition.

Study	Number participants	Methodology	Effect of iconicity
**L1 acquisition**
Orlansky and Bonvillian, 1984	13	Parental reports on children’s productive/receptive vocabulary	Null
Meier et al., 2008	4	Assesment of children’s exaggeration of iconic features of signs	Null
Ortega et al. (2014, 2017)	48 (20 children, 28 adults)	Production of action or perceptual sign in a spatial description	Preference for action signs
Anderson and Reilly, 2002	69 (34 for longitudinal data)	MacArthur Bates CDI (ASL)	Iconicity was not manipulated
Woolfe et al., 2010	29	MacArthur Bates CDI (BSL)	Iconicity was not manipulated
Rinaldi et al., 2014	8	Picture naming task (sign)	Iconicity was not manipulated
Thompson et al., 2013	31	MacArthur Bates CDI (BSL)	Positive
**L2 acquisition**
Lieberth and Gamble, 1991	50	Sign recall (in English)	Positive
Campbell et al., 1992	53	Forced-choice and naming task	Positive
Baus et al., 2012	30 (15 non-signers, 15 proficient signers)	English-ASL translation	Positive
Morett, 2015	26	Sign learning (production of signs)	Positive
Ortega and Morgan, 2015a	30	Sign repetition (imitation of signs)	Negative
Ortega and Morgan, 2015a	9 (longitudinal)	Sign repetition (imitation of signs)	Negative

### Degree of Iconicity and Sign L1 Acquisition

Recent advances on how motivated linguistic forms operate in different non-Western spoken languages can illuminate our understanding of how iconicity may have an involvement in L1 acquisition. One such advancement is the relationship between the gradient nature of iconicity and language acquisition. Linguists recognize that there are different types and degrees of word-referent mappings with some forms linking more directly to their referent than others (Dingemanse et al., 2015). Absolute iconicity, for instance, is understood as a one-to-one relationship between some aspects of the referent and a linguistic form; relative iconicity involves forms resembling different relations between meanings (Dingemanse et al., 2015). Words with absolute iconicity are more easily understood than other types of iconic words because they map more faithfully to the perceptual features of the referent. Akita (2013) puts forward a Lexical Iconicity Hierarchy (LIH) in which sound-symbolic words vary in their degree of iconicity with each sub-type having distinctive forms and functions in language. The LIH posits that iconic words with absolute iconicity are more often found across different languages and have specific phonemic and morphosyntactic distributions. Crucially, Akita (2009) found a clear order of acquisition with the most iconic words being mastered first (i.e., absolute iconicity) and the less iconic ones being mastered at later stages.

In sign language research, studies have shown that signs rated as highly iconic are the first to be acquired by deaf children (Thompson et al., 2013) but it remains an open question which are these signs and what are the iconic features that children exploit for vocabulary learning. Based on what has been observed in spoken languages (Akita, 2009, 2013) and on current theories of sign iconicity (Emmorey, 2014), it is possible to argue that action-based signs (i.e., structures with absolute iconicity) may be responsible for positive effects of iconicity observed in sign L1 acquisition. The notion of Iconicity as Structure Mapping (Emmorey, 2014) posits that a signed phonological representation may overlap in varying degrees with a conceptual representation. According to this view, effects of iconicity in language development and processing will be observed when sign and referent have high degree of overlap, for example, when signs represent actions (i.e., the hand represents the hand). This position would predict that deaf children will be biased to acquire signs representing actions because of the high degree of overlap between sign and referent (e.g., the sign TO-DRINK in many sign language has direct correspondences to the action of drinking). This prediction is supported by research showing that deaf children have a strong preference for predicates over nominals (Anderson and Reilly, 2002; Woolfe et al., 2010; Rinaldi et al., 2014); as well as action-based signs during production (Ortega et al., 2014, 2017) and comprehension tasks (Tolar et al., 2008). The close connection between real actions and action-based signs may aid the problem of referentiality (Perniss and Vigliocco, 2014) and in turn jump-start vocabulary development (Imai and Kita, 2014). The direct mappings between action and signs could also be beneficial at a stage when infants lack a fully developed phonological system. In the spoken modality, for instance, infants favor onomatopoeic forms instead of conventional words because they give them the opportunity to express ideas about a referent despite their limited phonological repertoire (Laing, 2014). In sign languages, action and action-based signs converge in many formational and semantic aspects so children may take advantage of these similarities to communicate while they develop a manual phonology.

Future research may investigate to what extent this type of sign iconicity facilitates lexical development and pave the way for other less iconic signs. Of course, iconicity alone cannot explain sign acquisition because parental input (Perniss et al., 2017) and children’s monitoring of signs’ properties are also exploited to scaffold learning (Caselli and Pyers, 2017). In addition, other factors that have shown to impact lexical development in speech may also play a role in sign acquisition, for example parents’ education (Woolfe et al., 2010), socio-economic status (Fernald et al., 2013), type of child-parent interaction (Bornstein et al., 2015), to name just a few. Understanding how these factors interact with certain types of iconicity will give a comprehensive picture of lexical development in deaf children.

### Iconicity Aids Semantic Aspects of Sign L2 Acquisition

A caveat in many studies investigating L2 acquisition is that lexical learning is often regarded as a monolithic piece of linguistic information when in fact signs and words consist of phonological and semantic representations. Many experimental paradigms have used reaction times, forced-choice tasks, and translations into participants’ first language as proxy of vocabulary learning. While these measures are good approximation of the emergence of *receptive* word knowledge they do not reveal entirely the psychological reality of lexical development. It is possible that a categorical approach toward sign learning can explicate the opposing findings of iconicity in sign L2 acquisition. The argument put forward here is that by teasing apart sign acquisition in its two constituents (i.e., form and meaning) one may see iconicity’s focus of influence in sign L2 learning.

Second Language studies have been pivotal in understanding how learners acquire the two components of new lexical items. There is strong evidence that there is often dissociation between the formal and conceptual aspects of word learning with meaning taking precedence over form (i.e., learners will look for meaning before worrying about the form of newly learnt words) (Barcroft, 2004, 2015). In addition, learners have limited cognitive resources and depending on the demands of a given task they will focus only on one aspect of the target word (VanPatten, 1990, 1996; VanPatten and Cadierno, 1993). For instance, when learners are instructed to carry out ratings of pleasantness on novel L2 words (e.g., which recruits deeper semantic processing) they perform better at tasks that evaluate knowledge of word meaning than tasks assessing its phonological form. When they are asked to make judgements about words’ phonemes (e.g., which recruits deeper phonological processing), they perform better at form-based than meaning-based tasks (Barcroft, 2004, 2015). Learners’ performance on L2 words will depend on what aspect of word learning they focus on and the task used to assess vocabulary learning.

In this review article it is argued that the positive and negative effects in sign L2 learning relates to some studies evaluating sign meaning and others sign form. On one hand, experiments showing a positive effect of iconicity implemented tasks that evaluated knowledge about the meaning of signs and not production of their exact phonetic structure (Lieberth and Gamble, 1991; Campbell et al., 1992; Baus et al., 2012)^2^. On the other hand, studies reporting a negative effect of iconicity are those exploring the actual phonetic articulation of lexical signs (Ortega and Morgan, 2015a,b). If we consider that the structure of iconic signs give away part of their meaning, one could argue that learners channel their cognitive resources to the conceptual/semantic aspect of the sign because it is more readily available. By default, individuals favor meaning over form at the initial stages of L2 learning (VanPatten, 1990), so increased semantic processing due to iconicity may deplete resources to learn a sign’s form. When signs have arbitrary mappings with the referent, L2 learners must develop some basic phonological representation to recognize them and consequently access their meaning.

The L2 evidence presented here suggest that iconicity has a positive influence in the conceptual/semantic aspects of sign learning but a negative or null effect in the acquisition of the actual linguistic form (similar findings have been reported on the effect of iconic gestures in L2 lexical acquisition; Kelly et al., 2009; Kelly and Lee, 2012). Future research should establish the locus of influence of our experimental design (i.e., form or meaning) so as to better understand how direct form mappings affect sign L2 acquisition. Implementing the form-meaning distinction experimentally brings important methodological hurdles given the lack of a clear notion of phonological complexity, degree of iconicity, and crucially, a standardized articulation coding scheme (although there have been some recent some advances on the latter; Ortega, 2013; Chen Pichler et al., 2016). However, the existing research and the developing technologies can serve as stepping stone toward unified and improved methodologies to investigate how hearing learners acquire the formal and conceptual/semantic aspects of signs.

## Iconicity Aid Sign Acquisition in Other Domains

Iconicity is a pervasive property observable not only at the lexical level but also in other linguistic structures such as signs’ phonological constituents (Stokoe, 2001; van der Kooij, 2002), morphological markers (Wilbur, 2003), and classifier constructions (Emmorey, 2003). Critically, the iconicity encoded in these different levels seems to be accessible to non-signers in different degrees. For instance, the temporality of signed verbs (i.e., telicity), which is iconically represented by means of repetitive movements (atelic) or abrupt end-points (telic), can be accurately differentiated by non-signers (Strickland et al., 2015). Given that this effect is observed in a large number of real and artificial sign languages, it may be possible to speculate that the capacity to link certain features of a sign (e.g., movement) with an abstract concept (e.g., telicity) may facilitate some aspects of sign learning.

**FIGURE 5:**
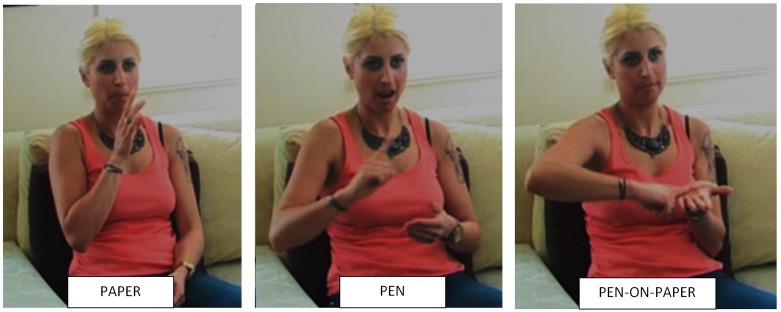
Classifier construction in Turkish Sign Language (*Turk Isaret Dili,* TİD). The lexical signs PAPER (ground) and PEN (figure) precede the structure PEN-ON-PAPER which represents iconically the spatial relationship between both objects.

Classifiers are another type of sign structures that may also be susceptible to the effect of iconicity in sign learning. Spatial descriptions in sign languages typically introduce the lexical items involved in a scene (i.e., ground and figure) followed by classifiers representing the physical properties of each referent. For example, in TİD the description *pen on paper* requires introducing the signs PAPER and PEN (i.e., ground and figure), followed by two classifiers representing their shape. The handshape 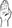 represents the flatness of a sheet of paper, the handshape 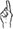 represents the thin elongated form of a pen, and the configuration 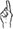 on 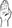 represents a thin object lying on a flat surface (**Figure 5**). These structures (also called proforms, depicting verbs, productive morphemes, and mimetic depictions) have been at the center of a heated debate over many years. While some researchers regard them as discrete morphemes comparable to classifiers in speech (e.g., Supalla, 1982; Aronoff et al., 2003; Sandler and Lillo-Martin, 2006) some others consider them manual forms with evident gestural features (e.g., Klima and Bellugi, 1979; Liddell, 2003; Schembri, 2003). Regardless of these two stances, it is inevitable to see that classifier constructions depict iconically multiple aspects of a spatial scene (e.g., physical attributes of the referents and their spatial distribution). Interestingly, recent research shows that iconicity facilitates acquisition to some extent.

The first studies investigating L1 acquisition reported that deaf children have great difficulty learning classifier constructions because these are highly complex morphological structures not mastered until after late in childhood (around the age of 9 years of age) (e.g., Kantor, 1980; Supalla, 1982). However, recent studies testing a significantly larger number of participants, and comparing directly child and adult production, show that deaf children as young as 3 years-old, are capable of producing adult-like classifier constructions (Sümer, 2015; Simper-Allen, 2016). Importantly, hearing children learning a spoken language lag behind deaf kids in the production of the same spatial descriptions because iconicity maps more directly to a linguistic form, than in speakers who use arbitrary terms for abstract concepts (e.g., prepositions or case markers) (Sümer, 2015). In the context of sign L2 acquisition, Marshall and Morgan (2015) report that hearing BSL learners struggle producing the canonical handshape associated to a referent with very high error rates (around 60%). However, iconicity has a positive effect in comprehension tasks because non-signers and hearing learners perform well above chance in tasks requiring them to match a signed spatial description with their referent. The authors argue that iconicity does not help in the acquisition of the linguistic conventions of classifier constructions (i.e., the form of the handshape) but it does help in the acquisition of the parameters that are more directly linked to real topographic space (i.e., location and orientation).

Together these studies show that iconicity may facilitate sign acquisition in other linguistic domains, but more studies are required to answer these new empirical questions. The ideas presented in this review could guide future research aiming to investigate how and where iconicity operates during the acquisition of other signed structures.

## Conclusion

This article has reviewed the literature of the evidence on the role of iconicity in the acquisition of a sign language by deaf children (L1) and hearing adults (L2). The chief objective was to reconcile the contradicting evidence and point at areas where the main discrepancies stem from. In a nutshell, these relate to the operationalisation of iconicity as a monolithic feature of signs and to the assumption that sign learning consists on the acquisition of a categorical lump of knowledge. When we appreciate (i) the gradient nature of iconicity and (ii) that signs consist of a phonological form attached to a meaning we can discern how iconicity modulates some aspects of sign acquisition in different populations.

The evidence presented here points to the tremendous need for a consensus on the operationalisation of sign iconicity. The extensive capacity to express iconic structures in the manual-visual modality places signed languages in a privileged position to answer questions about the human ability to communicate. Investigating the acquisition of motivated sign forms will further our understanding of sign languages and the finding will resonate in other disciplines interested in the ontogeny and phylogeny of language. Moving away from idyllic preconceptions of language, and probing the psychological reality of iconic signs in real use will be a major step forward in language and cognitive sciences.

## Author Contributions

GO was the sole contributor of this review article.

## Conflict of Interest Statement

The author declares that the research was conducted in the absence of any commercial or financial relationships that could be construed as a potential conflict of interest.

## Footnotes

1 There is an unsolved debate whether there is an intermediate level between semantic and phonological representations which encodes syntactic information about a word (Levelt, 1989; Levelt et al., 1999). I will ascribe to those authors arguing against it (Caramazza, 1997) and will not delve into the discussion as it falls out of the main aim of this review.

2 Note that even though the translation study (Baus et al., 2012) includes sign production, the authors used reaction times as proxy of sign learning but they did not directly investigate accuracy in sign articulation.
